# Effectiveness of a large-scale home visiting programme (PIM) on early child development in Brazil: quasi-experimental study nested in a birth cohort

**DOI:** 10.1136/bmjgh-2021-007116

**Published:** 2022-01-23

**Authors:** Eduardo Viegas da Silva, Fernando Pires Hartwig, Fernando Barros, Joseph Murray

**Affiliations:** 1 Postgraduate Program in Epidemiology, Federal University of Pelotas, Pelotas, RS, Brazil; 2 Human Development and Violence Research Centre (DOVE), Federal University of Pelotas, Pelotas, RS, Brazil; 3 State Health Surveillance Centre of Rio Grande do Sul, Porto Alegre, RS, Brazil

**Keywords:** health systems evaluation, child health, cohort study, epidemiology

## Abstract

**Background:**

A large-scale parenting programme with weekly home visits (Primeira Infância Melhor (PIM)) has been implemented in the south of Brazil for nearly two decades, but lacks evaluation of its effects on early childhood development (ECD). This quasi-experimental study aimed to assess the effects of PIM in real-life settings within a population-based birth cohort study.

**Methods:**

Data from the 2015 Pelotas Birth Cohort Study and the state programme information system were linked to identify study children who received PIM. Propensity score matching was used to create a comparable control group (using one-to-one matching) to estimate the effect of PIM on ECD measured at age 4 years. First, the impact of any enrolment in PIM was evaluated; then the intervention group was stratified according to whether enrolment occurred during pregnancy or after birth. Double adjustment was applied in linear regression to analyse child development scores, and Poisson regression for delayed development (below the 10th percentile of whole cohort). Effect modification due to family income was explored.

**Results:**

There was no evidence that any enrolment in PIM (601 pairs) by age 4 years was associated with child development. However, PIM starting during pregnancy (estimated for 121 pairs) predicted 60% lower prevalence of delayed development (prevalence ratio=0.40; 95% CI 0.18 to 0.89), compared with the control group. There was strong statistical evidence (p=0.02, test of interaction) that the effect of PIM starting in pregnancy was larger than when starting after birth (480 pairs). The effect of PIM starting during pregnancy was not modified by family income at birth.

**Conclusions:**

In a real-life setting, PIM was effective only when starting during pregnancy. A higher-quality programme might be more effective with a broader population.

**Key questions**

**What is already known?**

- Adequate early childhood development (ECD) is a strong predictor of school performance, adult income and physical and mental health.
- Vast numbers of children in low-income and middle-income countries (LMICs) do not reach their developmental potential.
- Researcher-implemented home-visiting programmes appear to have moderate to large effects on ECD in LMICs, but the effects of implementation at scale in real-life settings remain unclear.

**What are the new findings?**

- Based on a large Brazilian birth cohort, no overall effects on ECD were found of a large-scale home-visiting programme (Primeira Infância Melhor) starting anytime up to age 4 years in real-life settings.
- There was evidence for differential effects according to the timing of enrolment. Initiation during pregnancy was associated with improved ECD, but not initiation after birth.

**What do the new findings imply?**

- This study highlights the need for improved implementation and better targeting mechanisms to actively search for and include pregnant women most at need of support.

## Introduction

Adequate early childhood development (ECD) is essential to realise full human potential over the life course.^1^ It acts as a strong predictor of school performance, productivity, income and physical and mental health in adulthood.^2–6^ Given greater brain plasticity and sensitivity to environmental stimuli in the first 1000 days of life, supporting ECD in this period is a foundation of sustainable and thriving societies.^7^ However, it has been estimated that 43% of children under 5 years of age in low-income and middle-income countries (LMICs) are at risk of not reaching their developmental potential,^8^ and in many countries, there has been little or no progress in supporting improved development over recent years.^9^

There are marked socioeconomic differences in ECD both within and between countries,^10^ which is one key mechanism through which poverty and health inequities transmit across generations. Such development inequalities are already detectable in the first year of life, and then widen over the course of childhood.^11 12^ Reducing these gaps is, therefore, a primary aim of early interventions geared to support vulnerable families in providing nurturing care. Given the scale of the challenge in many LMICs, and lack of resources, there is a need for affordable interventions that can be delivered by non-professionals to support nurturing care and ECD at scale.

Evidence, mostly from researcher-implemented interventions in smaller-scale randomised clinical trials (RCTs), suggests that parenting programmes with home visits have a moderate to large effect on ECD in LMICs when rigorously implemented with an appropriate curriculum.^13–17^ On a larger scale, an RCT that evaluated the impact of an early childhood stimulation programme conducted in Bangladesh (N=2425) reported implementation challenges and showed an effect of 0.08 SD for cognitive development and 0.14 SD for language development, which were smaller than those found in other smaller-scale and higher-fidelity assessments.^18^ Some studies have suggested that the effects may be particularly pronounced when interventions are initiated in pregnancy, thereby increasing family engagement in the programme and supporting preparatory learning of parenting skills and parent–child attachment.^19–21^ However, the strength of evidence on the differential impact of initiation during pregnancy is low, as studies have not been designed primarily to answer that question. Moreover, rapid brain development for different sensory and cognitive systems extends well into the postnatal period.

The effects of real-world implementation of parenting programmes at scale have rarely been evaluated. Results from researcher-led interventions in RCTs are difficult to generalise, given the challenges of implementation with the same intensity and fidelity to content in real-world settings. Moreover, even when researchers have minimal involvement in intervention delivery and implementation, the presence of a research team tends to affect the rigour of assessment of eligibility criteria, training for service delivery and behaviour of programme managers,^22^ which means that the results may not precisely translate to real-world settings. Other factors, such as the ‘dose’ of intervention received by the target population, and its characteristics and environment, are also likely to differ in RCTs, compared with real-world conditions. Hence, high-quality quasi-experimental studies that allow evaluation of intervention effects under routine conditions are essential for considering the effectiveness of large-scale application of interventions. Their findings need to be combined with evidence from efficacy trials, for public health decision making.

A large-scale parenting home-visiting programme, the Primeira Infância Melhor (PIM), was implemented as public policy in the state of Rio Grande do Sul (southern Brazil) in 2003. In total, this programme has now supported over 240 000 children and 58 000 pregnant women, and is a model for the Criança Feliz home-visiting programme, now implemented throughout Brazil, which is one of the largest ECD programmes worldwide. However, there are still no published evaluations of the effect of PIM or Criança Feliz on ECD in quasi-experimental longitudinal studies or RCTs. Previous ecological studies suggested the existence of associations between PIM and reduced child mortality from external causes^23^ and reductions in school violence.^24^ One cross-sectional study with a sample of 571 children found no differences in ECD between PIM intervention and control groups.^25^ To support further planning of PIM and inform policies on similar interventions in LMICs, the current longitudinal, quasi-experimental study based on preintervention characteristics affecting selection into the intervention, and postintervention tests of ECD, aimed to assess the effects of PIM on ECD in real-life settings within a population-based birth cohort.

## Methods

### Design and participants

A quasi-experiment was conducted, nested in the 2015 Pelotas (Brazil) Birth Cohort Study. Pelotas is a city in southern Brazil, with around 340 000 inhabitants. All hospital-delivered children live-born in Pelotas between 1 January 2015 and 31 December 2015, whose mother lived in the urban area of the city, were eligible for inclusion in the study. Out of the 4333 eligible live births, 4275 were assessed at birth (response rate 98.7%). All these children and their mothers were invited to follow-up assessments at 3, 12, 24 and 48 months; the 4-year follow-up was conducted in a university research centre, while the earlier postnatal visits were conducted in the children’s homes. Further information about the 2015 Pelotas Birth Cohort is available elsewhere.^26^ At 4 years, when the main outcome data for the current analyses were collected, 4010 participants were assessed (mean age=3.8 ± 0.2 years), and 67 participants were identified as having died (giving a total follow-up rate of 95.3% of the original cohort).

Primary data from the cohort and secondary data from the state PIM information system were linked based on municipality (Pelotas), child’s date of birth, child’s name and mother’s name. The data collected from the PIM information system were age at admission, length of stay in the programme, reason for withdrawal from PIM, number of different visitors who accompanied the child (carried out home visits) throughout their participation in the programme, and involvement of any older sibling in the programme. PIM funding depends on the number of children registered by the municipality, so it is unlikely that children receiving the intervention were not registered.

The mothers and interviewers were blind to the hypotheses of this study. The assessors of child development were not aware of the child’s participation in PIM, or of the aim of the current study to evaluate the impact of PIM. The linkage of databases was carried out without any involvement of the assessors who collected information about the outcome and the potential confounders at cohort follow-ups.

### Intervention

PIM was first implemented in Pelotas in 2003, and is managed by the Municipal Health Department with direct support from the State Health Department, which developed the programme. It aims to enhance sensitive and responsive caregiver-child interactions through engagement in age-appropriate play activities, along with provision of information for nurturing care and facilitating access to health and social services. Weekly home visits (45–60 min) are made by trained health, education and social science undergraduate students, selected by the health authority to work with children and their caregivers in this role; additional group activities are used with 3–5 years. Visitors are selected via a process including an initial 60-hour training period (selection takes place partly during this training period), and then selected visitors receive ongoing weekly training. Each visitor serves a maximum of 17 families with a maximum weekly workload of 30 hours. They receive scholarship support in this role from the Municipal Health Department (not from their university), for a maximum duration of 2 years. They are directly supervised by a monitor responsible for up to eight visitors, with whom they discuss the families’ care plan based on programme materials and conduct joint home visits using an observation guide to assess the quality of the visit and provide feedback. The monitors are visitors who, after a period of fieldwork with families, are identified by the municipal management group as having a profile for team leadership, and are hired with the same type of contract as the visitors. Home visits involve listening to the family, reviewing the activities of the last week, conducting a playful activity aimed at stimulating child development and planning activities for the next week. Information on child health and nutrition is also provided, and referrals to healthcare services or social assistance are made as necessary. Routine assessments of the child’s development are made every 3 months.^27^

PIM focuses on families with greater social vulnerability, although no objective eligibility tool was used. The families included were those identified by visitors and municipal staff as vulnerable during day-to-day work, or those indicated by healthcare services or even by previously assisted families. Inclusion also depended on resources available and the family’s agreement to participate. The families of children enrolled in PIM were mostly of low socioeconomic status: 34% and 29%, respectively, belonged to the poorest and second poorest quintiles of family income at birth. Additionally, 54% of PIM mothers had eight or less years of education, compared with 27% of the mothers of children who did not receive the intervention (online supplemental table 2). Nonetheless, from a population perspective, PIM was not applied to all those who would be considered to be priorities for programme eligibility: 67% of all the children in the cohort in the lowest quintile of family income at birth were not included in PIM. The relatively low coverage of the programme among those in need made it possible to apply the quasi-experimental method used in this study.

10.1136/bmjgh-2021-007116.supp1 Supplementary data

We examined the intervention defined in two different ways according to timing of enrolment. First, we examined the impact of any enrolment in PIM: all children enrolled in the programme from any age up to 4 years were considered as having participated in PIM. Second, we examined the impact of participating in PIM according to whether families were enrolled during the mother’s pregnancy (with the focal study child) or after birth.

### Outcome

Child development was measured at age 4 years using the screening version of Battelle’s Developmental Inventory (BDI). This instrument consists of 96 items divided in five domains of neurodevelopment (personal-social, adaptive, motor, communication and cognitive) for children ranging in age from birth to 8 years.^28^ This had previously been translated to Brazilian Portuguese and was adapted to form a reduced 66-item instrument (using all items for each age level from birth to 4–5 years of age, but excluding items for older ages).^29^ BDI was applied by trained interviewers who were supervised by child development psychologists. The instrument was divided into 13 questions for the mothers (applied first) and 53 items or fewer that were directly applied to or observed among the children (without the mother’s presence in the room). After applying the items assessing milestones for children aged 4–5 years and 3–4 years, which are unconditionally applied to all children, the evaluation continued with application of items relevant to younger ages (first ages 2–3 years, then younger). The evaluation (each domain) terminated when the child achieved the maximum score (2) for two consecutive items. At that point, items referring to lower-difficulty (younger age) skills were automatically scored as two points.

A total development score ranging from 0 to 132 and scores for each subdomain were standardised based on their distribution in the study sample. Children with a development score <50 were excluded because they were considered to have a severe mental deficit. The total score (after exclusion of children scoring <50) was also dichotomised to define a group with low developmental score, using the cut-off point for the 10th percentile; this identified children whose developmental score did not surpass that expected of children aged 30 months.

For age 4 years, the BDI instrument presents good validity for predicting later development.^30^ Quality control was performed in the current study for 200 randomly selected children, through use of videos recording the application of the instrument to the child. The total score calculated, based on the coding by trained interviewers, showed strong agreement with the total score calculated from coding by senior psychologists who observed the videos, taking into account the application environment, the interviewers' approach and the children’s responses (kappa statistics indicated strong or excellent agreement for all the domains analysed).

### Statistical analysis

After linking databases and identifying children in the cohort who received the PIM programme, propensity scores (PS) were calculated^31^ for the probability of participation in PIM. Subsequently, we matched each child who received PIM to one participant from the pool of potential controls based on their PS, to subsequently estimate the effect of PIM on child development. Altogether, 27 covariates (details of measurements are presented in online supplemental box 1) were used in order to estimate the PS using logistic regression, in which participation in PIM was the dependent variable. First, covariates weakly associated (p<0.20) with both the intervention and the outcome were used to estimate a preliminary PS and balance was examined. Any additional covariates that were imbalanced after this initial matching were then included in a new logistic regression model (along with the originally included covariates) to improve balance. Individuals with missing data for any covariate used in this logistic model were excluded from the analytical sample, given imputation methods would be difficult to operationalise in paired analysis with double adjustment (further adjustment of covariates in final estimates of the effects of PIM on study outcomes). The covariate with the highest percentage of missing data (couple’s relationship quality: 17.5%) was not included in the PS calculation, in order to reduce losses.

All potential confounders were measured from maternal reports during the perinatal assessment, except for the following: main caregiver until the child reached 3 months of age; depressive maternal symptoms and the couple’s relationship quality, which were measured at the 3-month assessment; childcare attendance, which was measured at the 2-year assessment; and neighbourhood violence, which was measured at the 4-year assessment. Having an older sibling who had received PIM was included as a covariate to reduce residual confounding, since families previously involved in the programme, but whose study child (from the 2015 birth cohort) did not receive the intervention, were considered to provide robust controls against self-selection bias.

PS distribution curves were compared for groups that received and did not receive PIM, for an initial assessment of the plausibility of adequate matching. PS matching was performed without replacement, starting with individuals in the intervention group with the highest PS value. After matching, covariate balance was assessed for all 27 covariates, considering a standardised mean difference of 0.1 as a maximally acceptable difference between groups.^32^

Analyses of the BDI score were based on linear regression. Analyses of the dichotomous indicator of ‘belonging to the group below the 10th percentile’ used Poisson regression for direct estimation of prevalence ratios.^33^ In both analyses, the matched pairs were treated as clusters, through the ‘survey’ command. This allowed straightforward additional adjustment for covariates in the outcome regression, thus enabling ‘double adjustment’ for covariates included as predictors in estimating the PS, which were all prognostically important factors. In view of the matching process, the results can be interpreted as the mean causal effect of the PIM in the intervention group (ie, the causal effect in the treated group).

We initially analysed any enrolment in PIM, to evaluate the whole programme as implemented in the population (including families enrolled at any time between pregnancy and child age 4 years). Afterwards, the intervention group was stratified according to whether families enrolled in PIM during or after pregnancy, and separate effects were estimated for these different enrolment times. For these stratified analysis, children who did not received PIM were randomly divided proportionally to the number of individuals in each intervention subgroup (PIM starting during or after pregnancy). Covariate balance between the two randomly generated non-PIM groups was examined to assess their interchangeability. For each stratum of the intervention group (enrolled during pregnancy or after birth), the steps described above for matching and analysis were conducted separately. For the stratum with initiation of PIM after birth, the same set of 27 covariates was used in the analysis. For the stratum starting during pregnancy, 15 covariates were selected for analyses. These were all potential confounders that were not on the causal pathway of the effect of PIM starting during pregnancy on ECD (online supplemental box 1). Cochran’s Q heterogeneity χ^2^ test was used to examine modification of the effect of PIM according to the timing of intervention initiation.

To investigate the programme’s potential to reduce inequalities in child development, effect modification according to family income quintile at birth was explored in double-adjusted analysis with low BDI (below the 10th percentile) as an outcome—in situations where significant main effects were found in the primary analyses. All analyses were conducted using Stata V.15.1.

### Patient and public involvement

The public was not involved in the design or conduct of our research. The municipal and state management of the PIM was involved in the planning of this evaluation. The results are being disseminated and discussed with those responsible for implementing the programme, to improve its impact.

## Results

**Figure 1:**
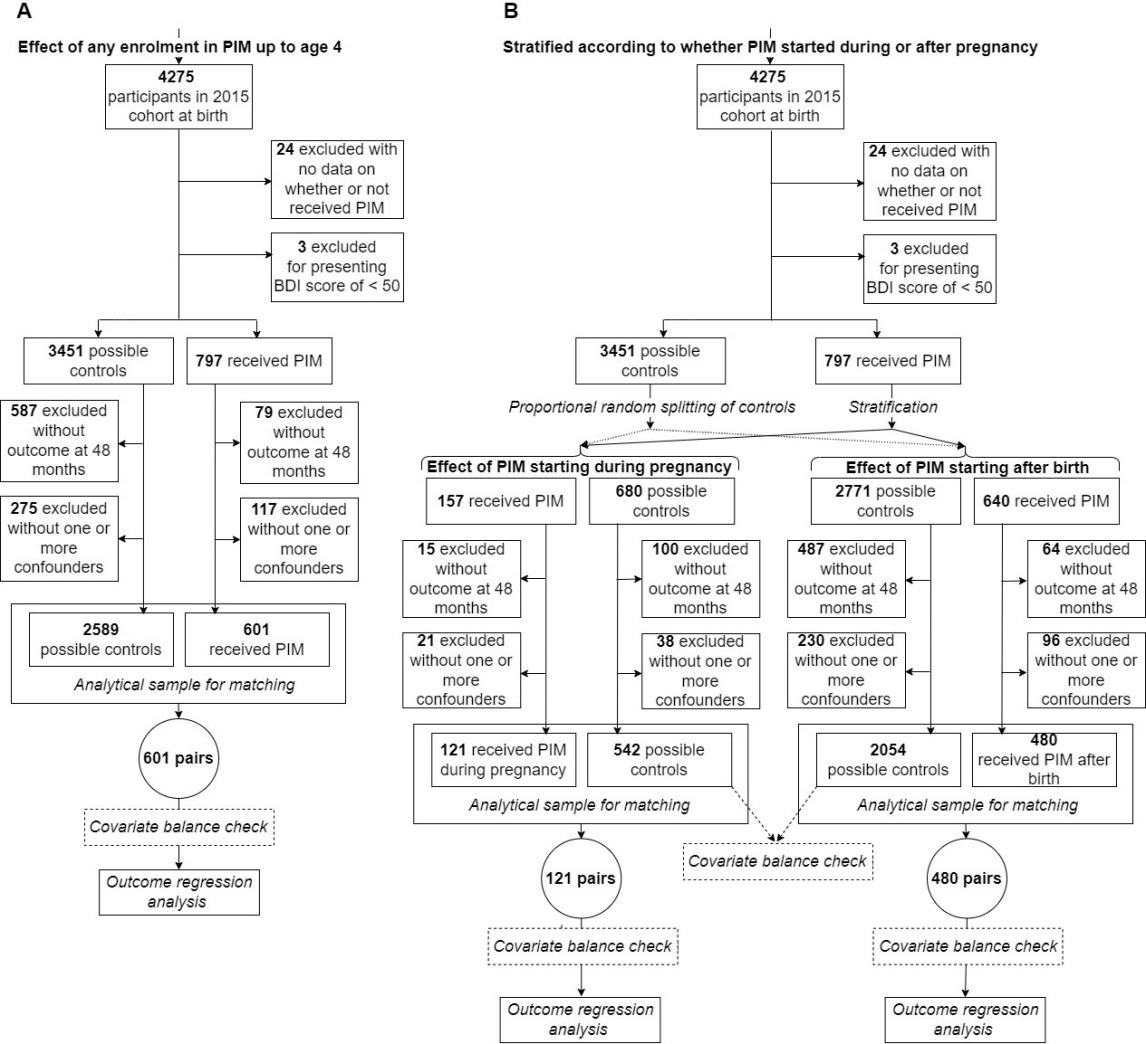
Flow chart showing numbers of children for whom propensity scores were calculated, numbers of children in matched analyses and reasons for exclusion, separately for estimation of both the effect of any enrolment in Primeira Infância Melhor (PIM) (A), and the effect of PIM stratified according to whether enrolment occurred during or after pregnancy (B). BDI, Battelle’s Developmental Inventory.

Out of 4275 children in the cohort, 797 were enrolled in PIM at any point up to their fourth birthdays. Of the whole cohort, 3190 children (74.6%) were included in the analytic sample, of whom 601 were enrolled in PIM. The one-to-one matching process based on PS was performed using that sample. Figure 1 presents a flow chart of the study analyses, which shows that most exclusions from the analytic sample were due to lack of outcome or covariate data. The excluded sample had somewhat lower socioeconomic status, but exclusions were not associated with receiving PIM (online supplemental table 1). Pregnancies were not planned by the family in 49% of the cases and 13% of mothers were under 20 years of age at birth (online supplemental table 1). Among children enrolled in PIM, 53% were enrolled up to age 12 months, and the duration of receiving the programme ranged from 3 to 42 months with a median of 12 months. The main reasons registered for withdrawal from the programme were lack of an available visitor (34%) and the family choosing to leave (25%) (online supplemental table 9). A high turnover of visitors for each child was recorded: among the 354 children enrolled in PIM at any time up to age 4 years and remaining in the programme for 12 months or more, 66% received the intervention from two or more visitors, and 65% of those 91 children who remained in the programme for 24 or more months received it from three or more visitors (see also online supplemental table 10). The total BDI score ranged from 56 to 131 points (mean=113.4; SD=8.8). Children with scores below the 10th percentile had scores less than or equal to 103 points, which is equivalent to a developmental age of 30 months, although the children were aged, on average, 46 months at assessment.

### Estimation of effects of any enrolment in PIM

**Figure 2:**
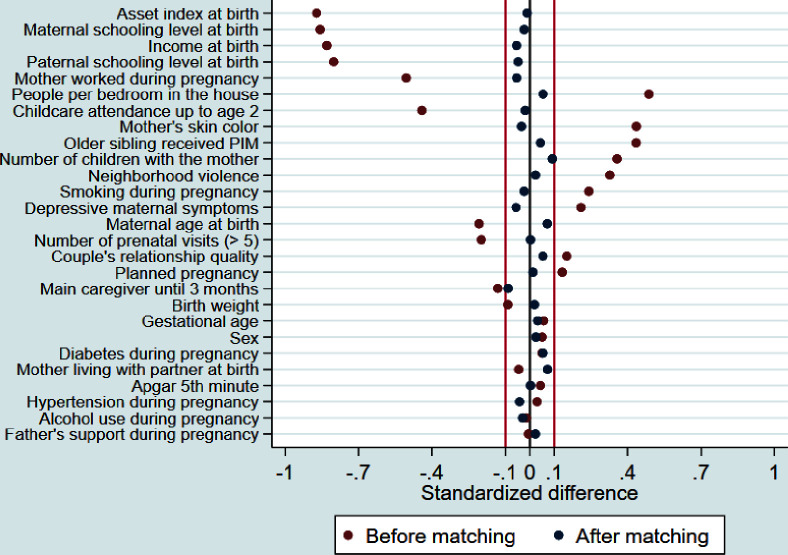
Standardised mean differences for potential confounders before and after propensity score matching in the analysis on any enrolment in Primeira Infância Melhor (PIM).

Figure 2 compares standardised mean differences of 27 confounders between PIM children (enrolled at any time) and the pool of potential controls (before PS matching, red dots in Figure) and matched controls (after PS matching, blue dots in Figure). The distribution of the PS for the PIM and potential control group (online supplemental figure 1) enabled matching of almost all PIM children to a control with a very similar PS, although for a few PIM children with very high PS, only controls with slightly lower PS were available. After matching, standardised mean differences between the PIM and the comparison group were less than 0.1 for all 27 covariates (see also online supplemental table 3).

Comparing PIM children (enrolled at any time) with matched controls, there was not strong statistical evidence that PIM affected child development measured at age 4 years (table 1). For example, with double adjustment, the effect of PIM on the total BDI score was estimated as almost exactly zero (β=0.02; 95% CI −0.09 to 0.13).

**Table 1:** Effects of any enrolment in PIM up to age 4 years (601 pairs) on child development at age 4 years

Outcome	Unadjusted*		Matched†		Matched with double adjustment‡	
	N=3190		N=1202		N=1202	
	β	95% CI	β	95% CI	β	95% CI
Linear regression for mean BDI score						
Total BDI score (SD)	−0.19	−0.28 to −0.11	−0.00	−0.11 to 0.11	0.02	−0.09 to 0.13
Personal-social (SD)	−0.17	−0.25 to −0.08	−0.03	−0.13 to 0.07	−0.02	−0.12 to 0.08
Adaptive (SD)	0.07	−0.01 to 0.16	0.05	−0.05 to 0.15	0.06	−0.04 to 0.16
Motor (SD)	−0.07	−0.16 to 0.02	0.02	−0.09 to 0.13	0.03	−0.08 to 0.14
Communication (SD)	−0.19	−0.28 to −0.11	0.03	−0.08 to 0.13	0.03	−0.07 to 0.14
Cognitive (SD)	−0.28	−0.37 to −0.20	−0.04	−0.15 to 0.08	−0.03	−0.14 to 0.09
Poisson regression for belonging to the group below the 10th percentile of BDI						
	**PR**	**95% CI**	**PR**	**95% CI**	**PR**	**95% CI**
Low development score	1.35	1.06 to 1.73	0.97	0.72 to 1.32	0.98	0.73 to 1.31

*Comparison between intervention group (n=601) and possible controls group (n=2589) without adjustment.

†Paired analysis comparing the intervention group (n=601) with the matched control group (n=601).

‡Paired analysis comparing the intervention group (n=601) with the matched control group (n=601) with double adjustment for the following confounders included in the propensity score prediction: neighbourhood violence, mother’s skin colour (others/white), maternal age at birth (>19 years), maternal schooling level (years), paternal schooling level, income at birth (quintiles), asset index at birth (quintiles), people per bedroom in the house, number of prenatal visits (>5), number of children with the mother, mother living with partner, mother worked during pregnancy, father’s support during pregnancy, planned pregnancy, depressive maternal symptoms (3 months), birth weight (grams), smoking during pregnancy, main caregiver until age 3 months, childcare attendance up to age 2 years and older sibling received PIM.

BDI, Battelle’s Developmental Inventory; PIM, Primeira Infância Melhor; PR, prevalence ratio.

### Effect modification of PIM according to enrolment during or after pregnancy

Next we compared the effects of PIM between families who were enrolled during pregnancy and those enrolled afterwards, in tests of interaction. This necessitated matching separately for each group to estimate the effects in each of them separately. Considering the 480 children who were enrolled in PIM after birth, matching yielded a good balance for all 27 covariates (online supplemental figure 4 and table 5)). For the 121 children whose families were enrolled in PIM during pregnancy, matching yielded good balance for most but not all covariates (online supplemental figure 6 and table 6), thus emphasising the need for double adjustment when analysing the effect of PIM starting during pregnancy.

In double-adjusted analysis, the test of interaction effect based on intervention timing (enrolment during or after pregnancy) was p = 0.08 for the overall child development score and p = 0.02 for low child development score, that is, below the 10th percentile. PIM starting during pregnancy was associated with 0.19 SD (95% CI -0.02 to 0.40) higher development scores at age 4, and with 60% lower prevalence (prevalence ratio=0.40; 95% CI 0.18 to 0.89) of having a low development score, that is, below the 10th percentile. In contrast, for PIM starting after birth, there was no strong statistical evidence of association with child outcomes (table 2). Exploratory analysis on the five separate domains of child development is presented in the online supplemental material, showing consistently larger effects across all domains, except the adaptive domain, when children were enrolled during pregnancy, with the cognitive domain presenting the strongest evidence for effect modification (online supplemental table 7).

**Table 2:** Effects of PIM on child development modified according to whether it started during pregnancy (121 pairs) or after birth (480 pairs)

Outcome	Unadjusted*				Matched†				Matched with double adjustment‡				
	Started during pregnancy		Started after birth		Started during pregnancy		Started after birth		Started during pregnancy		Started after birth		Heterogeneity p-value§
	N=663		N=2534		N=242		N=960		N=242		N=960		
	β	95% CI	β	95% CI	β	95% CI	β	95% CI	β	95% CI	β	95% CI	
Linear regression for mean BDI score													
Total BDI score (SD)	−0.07	−0.27 to 0.14	−0.22	−0.32 to −0.13	0.17	−0.06 to 0.41	−0.06	−0.18 to 0.07	0.19	−0.02 to 0.40	−0.03	−0.15 to 0.10	0.080
Poisson regression for belonging to the group below the 10th BDI percentile													
	**PR**	**95% CI**	**PR**	**95% CI**	**PR**	**95% CI**	**PR**	**95% CI**	**PR**	**95% CI**	**PR**	**95% CI**	
Low development score	0.76	0.39 to 1.50	1.50	1.15 to 1.96	0.45	0.20 to 1.00	1.14	0.82 to 1.60	0.40	0.18 to 0.89	1.12	0.81 to 1.54	0.020

*Comparison between intervention group and possible controls group without adjustment.

†Paired analysis comparing the intervention group with the matched control group.

‡Paired analysis comparing the intervention group with the matched control group with double adjustment for the following confounders included in the propensity score prediction for starting during pregnancy: neighbourhood violence, mother’s skin colour (others/white), maternal age at birth (>19 years), sex, maternal schooling level (years), paternal schooling level, income at birth (quintiles), asset index at birth (quintiles), people per bedroom in house, number of children with the mother, mother living with partner, mother worked during pregnancy, father’s support during pregnancy, planned pregnancy and older sibling received the PIM; and the following confounders for starting PIM after birth: neighbourhood violence, mother’s skin colour (others/white), maternal age at birth (>19 years), sex, maternal schooling level (years), paternal schooling level, income at birth (quintiles), asset index at birth (quintiles), number of prenatal visits (>5), people per bedroom in house, number of children with the mother, mother living with partner, mother worked during pregnancy, father’s support during pregnancy, planned pregnancy, depressive maternal symptoms (3 months), birth weight (grams), smoking during pregnancy, alcohol use during pregnancy, maternal hypertension during pregnancy, maternal diabetes during pregnancy, gestational age (<37 weeks), main caregiver until age 3 months, childcare attendance up to age 2 years and older sibling received the PIM.

§Cochran’s Q heterogeneity χ2 test.

BDI, Battelle’s Developmental Inventory; PIM, Primeira Infância Melhor; PR, prevalence ratio.

### Effects of PIM starting during pregnancy, according to family income at birth

Considering the effects of PIM starting during pregnancy on child development, and the well-documented social inequalities in child development, we explored possible effect modification due to family income. Such analysis should be interpreted with caution, given the small size of the sample of children who were enrolled during pregnancy. Exploratory analysis did not find evidence that family income at birth modified effects of PIM starting during pregnancy on the prevalence of low child development score (p = 0.44 considering income divided in quintiles, p = 0.60 considering income divided at the median).

## Discussion

In a large, population-based birth cohort study in southern Brazil, no effect of the PIM home-visiting programme on ECD was observed, considering all families who received the programme as one group. However, for families who were enrolled during pregnancy, there was a large decrease in the prevalence of having a low development score (below the 10th percentile). Given that the programme targeted more vulnerable families of low socioeconomic status, such benefits indicate potential for the programme, when starting in pregnancy, to reduce inequalities in ECD.

The lack of strong evidence for an effect of PIM in general (no effects observed for families enrolled at any time up to child age 4 years), contrasts with results from meta-analyses^13–17^ and single RCTs. These showed that home-visiting programmes had moderate overall effects on ECD in LMICs (of around 0.3 SD).^34–36^ However, as more programmes are scaled up, programmes with large coverage such as PIM may show null effects, given the challenges of high-quality implementation, requiring building bonds with the families involved, standardised high-quality content needing to be delivered, and achieving appropriate intensity of intervention.^1 37 38^

The challenges in implementing PIM include difficulties in establishing visitor-family bonds, as indicated by the relatively high visitor turnover recorded, and the fact that the main reasons for withdrawal from the programme were nonavailability of a visitor and the family choosing to leave. Also, half of the children were enrolled only after 1 year of age, even though the programme aims to promote ECD from the time of pregnancy onwards. This showed that the programme had relative low capacity to search for and engage families at the time of gestation and early infancy. Other studies have shown that staff with better knowledge of child development after initial training tend to deliver higher quality and more engaging content, which is associated with better results for parenting and ECD.^39^ Although PIM involves intensive initial and continuous weekly training,^27^ potentially reducing turnover^40^ and increasing the proficiency and confidence of the visitors with regard to delivery of the intervention,^39^ the fact that PIM visitors in Pelotas are undergraduate students with high turnover may have affected the level of social competence and ability to engage families in the programme curriculum. Compared with community health workers, undergraduates may have more theoretical knowledge, but less experience in parenting. Further evaluation of the effectiveness of different supervision strategies of the visitor is also important^41^; in PIM it is carried out by a monitor with previous experience as a visitor in PIM.

Despite the null results for the overall programme, we identified a positive effect of PIM home visiting on ECD when families were enrolled during pregnancy. Although the literature is sparse regarding differential effects according to whether visits start during pregnancy, our results corroborate those of several other studies.^20 21^ We can postulate three main possible explanations for this specificity of effect. First, pregnancy is an optimal time for establishing good relations between an expectant mother and a service provider. This is a critical period for fostering understanding of parenting issues,^42^ and promoting responsive caregiving and parent–child attachment, and this can be consolidated through continuity of the intervention after birth.^7 14 43 44^ Second, the strong emphasis of PIM on linking families with primary healthcare services, so as to promote prenatal care, may also have contributed, for example, via effects on birth weight. The explicit focus of the programme content on breastfeeding, which has been correlated with cognitive outcomes,^45 46^ may be another mechanism. Attachment developed from pregnancy onwards is a very consistent effect of parenting programmes^15^ and could have a synergistic relationship with breastfeeding promotion. A third possible explanation why starting PIM during pregnancy was associated with an effect that was not observed when PIM started after birth is that it simply led to longer overall participation in the programme. Among the children enrolled during pregnancy, about half received the programme for 18 months or more, while this was true for only about one quarter of the children who were enrolled after birth (online supplemental table 9). However, there was no clear pattern of association between duration of participation in the intervention and ECD outcomes, and there was no evidence of any interaction between participating in PIM (either starting during pregnancy or after birth) and the length of time receiving the programme, in predicting ECD (online supplemental tables 12 and 13). This is possibly because of the higher turnover of visitors for children with longer periods enrolled in the programme.

The apparently homogeneous effects of PIM starting during pregnancy across different family income strata contrasts with evidence from the same population showing that child stimulation influences child development with stronger effects among families with lower levels of education,^29^ and other intervention studies which have found stronger effects among less advantaged children.^15 20 47–49^ However, the literature is not entirely consistent, in that some studies have identified larger benefits for less vulnerable children.^34 50^

The potential of PIM and other similar interventions starting during pregnancy to improve ECD and reduce inequalities indicate: (1) the need for better targeting mechanisms to actively search for and include pregnant women in such programmes and (2) further research on whether post-natal home visiting can be made more effective through improved implementation, or content modification. To improve the focus of an intervention such as PIM, it is crucial that the managers of the programme should work with existing platforms such as primary healthcare and use prioritisation tools with high predictive validity to identify the families most at need of support.

One important limitation of this study was its lack of randomisation to treatment and control conditions. Despite extensive covariable adjustment, residual confounding may still have been present, and it is unclear whether this would be more likely to result in overestimation or underestimation of intervention effects. There was also a lack of detailed information on the quality of the PIM implementation, such as the number of visits received. Additionally, estimates of inequality reductions following enrolment in PIM during pregnancy were based on a relatively small subsample of the study. However, the study also had singular strengths in terms of evaluating the effectiveness of a large-scale home-visiting programme in an unconditioned real-life setting, without any interference from the study team regarding eligibility criteria or other aspects of the intervention implementation. Longitudinal measurements of a robust set of confounders were made, with a high level of accuracy, and any measurement error is likely to have been non-differential, given the blinding of assessors and participants to the study hypotheses.

## Conclusion

This study suggests that a large-scale home-visiting programme starting in pregnancy could improve ECD under real-world conditions in an LMIC setting. Given that the programme targets vulnerable poorer families, any effects have the capacity to reduce inequalities in ECD. However, the study also highlights an urgent need to improve the implementation process of the PIM programme. This is because no effect was observed for families who enrolled after pregnancy. The results from this study are directly relevant to the Brazilian context, but also speak to an urgent need for evaluation of large-scale implementations of home-visiting programmes delivered by paraprofessionals in LMICs, which are key to progress on target 4.2 of the sustainable development goals, that is, that all girls and boys should have access to quality ECD and care.^51^

## Data Availability

Data are available on reasonable request. Data are available on request. Due to confidentiality restrictions relating to the ethics approval for this study, no identifying information about participants may be released. The dataset without identification that was used during the current study is available from the corresponding author on reasonable request.
