# Correction: Effectiveness of a large-scale home visiting programme (PIM) on early child development in Brazil: quasi-experimental study nested in a birth cohort

**DOI:** 10.1136/bmjgh-2021-007116corr1

**Published:** 2022-05-04

Viegas da Silva E, Hartwig FP, Barros F, *et al*. Effectiveness of a large-scale home visiting programme (PIM) on early child development in Brazil: quasi-experimental study nested in a birth cohort. *BMJ Global Health* 2022;7:e007116. doi:10.1136/bmjgh-2021-007116

This article has been corrected since it was published online because of errors in the syntax of the STATA command that had been used in the matched models of the effects of the PIM intervention, which applied to many tables of results in the original publication. The syntax error related to the survey command in the regression analyses. We should have used the command “svyset pair_variable” and we previously wrongly applied “svyset [pw=pair_variable]”. Unfortunately, this had weighted the pair-identifier variable, rather than appropriately treating the pairs as clusters - the intended method. This and all other aspects of the statistical analyses and commands used to run them have been carefully reviewed in duplicate for the corrected version.

Unfortunately, the mistake in the syntax had consequences for the results as originally published, and specific aspects of the discussion. Having re-run the analyses with the correct STATA suffix, to appropriately adjust for clustered data, the two main conclusions of the paper persisted and remain in the corrected version of the article: (1) there are no effects of the PIM home-visiting programme on Early Childhood Development (ECD) considering all families who received the programme as one group; and (2) a positive effect on ECD is identified among families who were enrolled in PIM during pregnancy. However, previous results from exploratory analysis of moderation of the effects of PIM starting during pregnancy by family income at birth (originally published as significant based on the incorrect command) are not supported in the corrected results. Given the latter change in findings, a figure that was included in the original publication, displaying the interaction, has been removed from the corrected version now published online.

The current online version of the paper now presents corrected results, as well as revised interpretations of their findings where appropriate.

