# Supplementary material for: Effectiveness of a large-scale home visiting programme (PIM) on early child development in Brazil: quasi-experimental study nested in a birth cohort
Source: BMJ Glob Health. 2022 Jan 23;7(1):e007116. doi: 10.1136/bmjgh-2021-007116 (PMC8788193; doi:10.1136/bmjgh-2021-007116)
Supplement: Supplementary data [file bmjgh-2021-007116supp001.pdf]

Supplementary file:

Viegas da Silva E, Hartwig FP, Barros F, et al. Effectiveness of a large-scale home visiting programme (PIM) on early child development in Brazil: quasi-experimental study nested in a birth cohort. BMJ Global Health 2022;7:e007116. doi:10.1136/ bmjgh-2021-007116

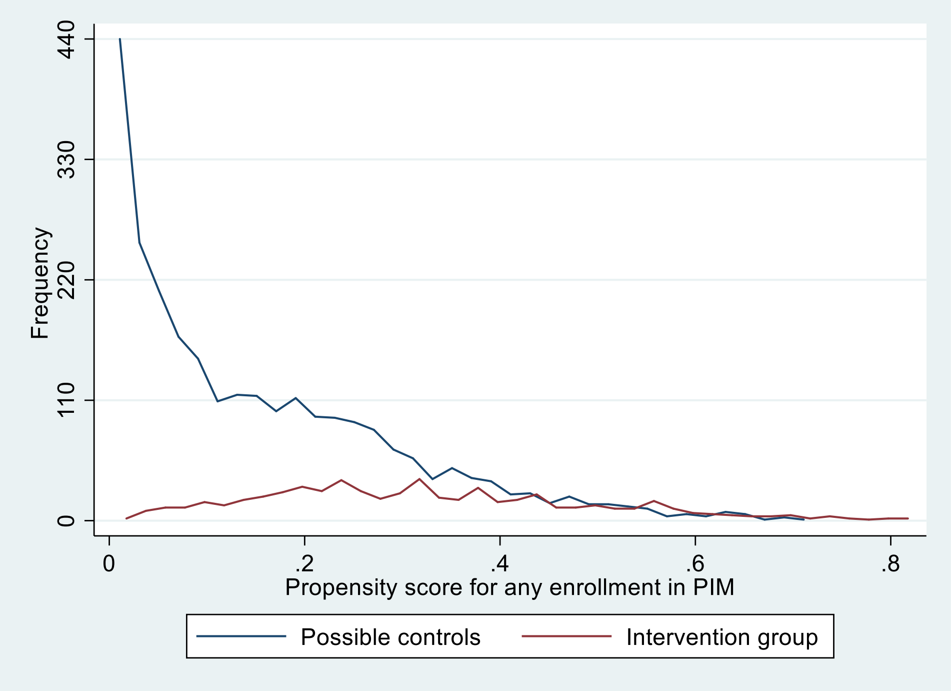

Supplemental Figure 1. Distribution of propensity score for intervention and control groups for analysis of effects of Primeira Infância Melhor (PIM) enrollment any time up to age 4 years.

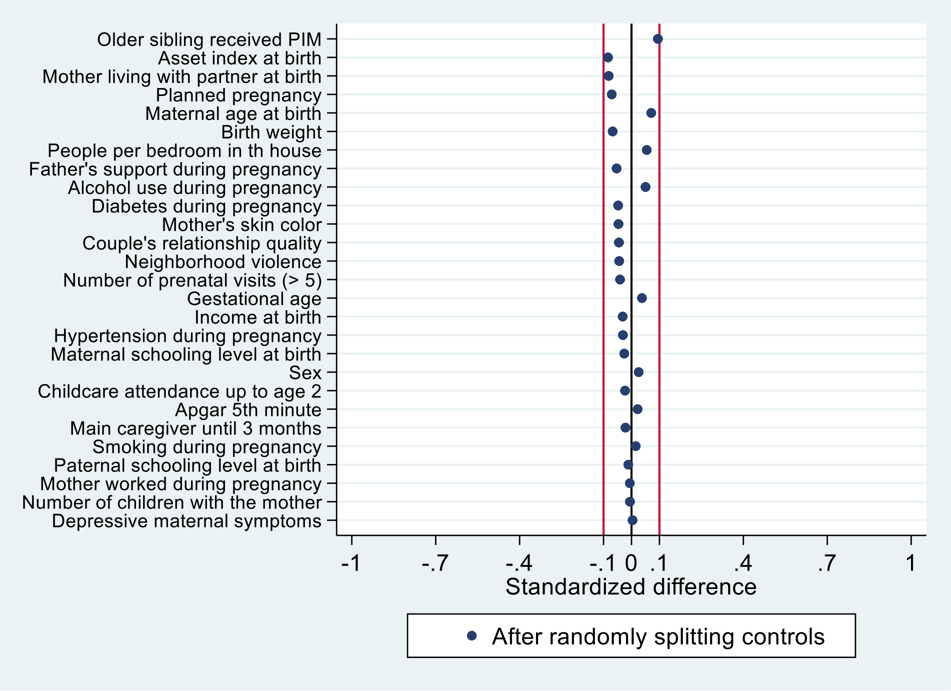

Supplemental Figure 2. Balancing of confounders between two randomly generated control groups used to test interaction with starting Primeira Infância Melhor (PIM) during pregnancy or after birth.

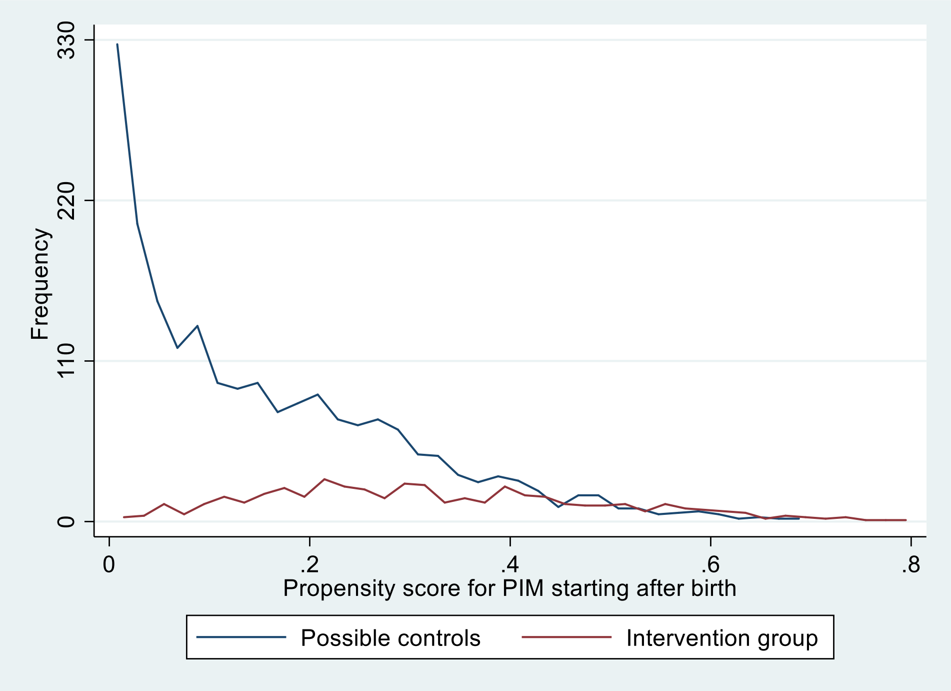

Supplemental Figure 3. Distribution of propensity score for intervention and control groups for analysis of effect of the Primeira Infância Melhor (PIM) starting after birth.

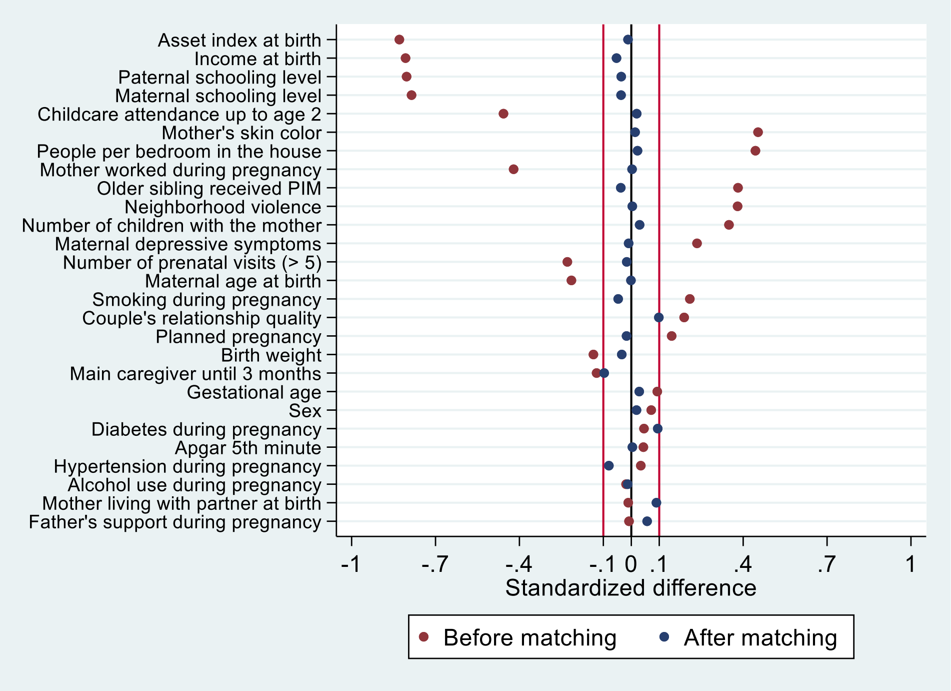

Supplemental Figure 4. Balancing of potential confounders before and after the propensity score matching that was used in the analysis on the effect of Primeira Infância Melhor (PIM) starting after birth (480 pairs).

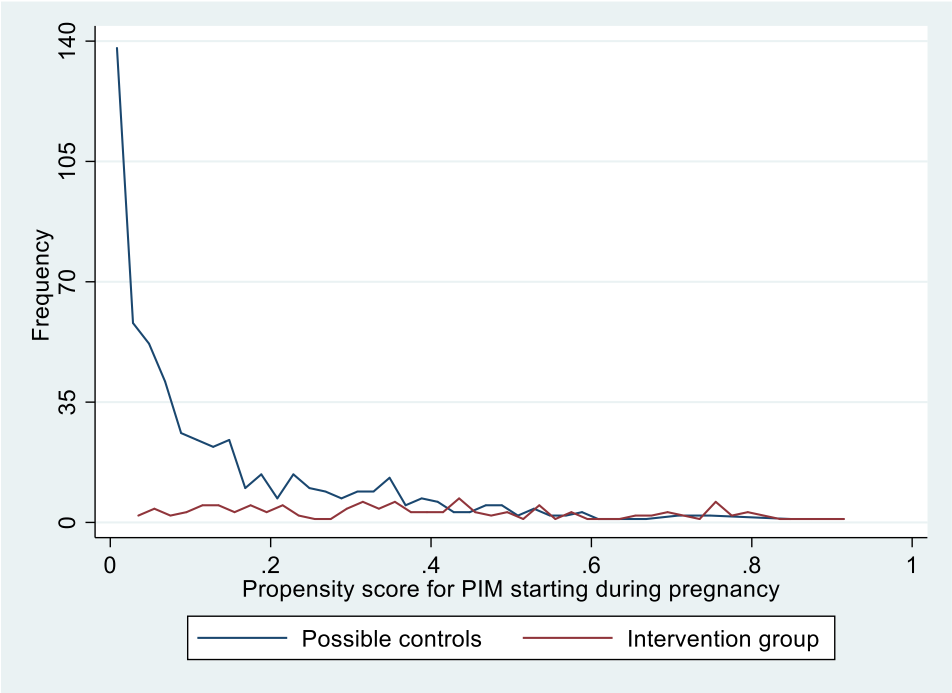

Supplemental Figure 5. Distribution of propensity score for intervention and control groups for analysis of effect of the Primeira Infância Melhor (PIM) starting during pregnancy.

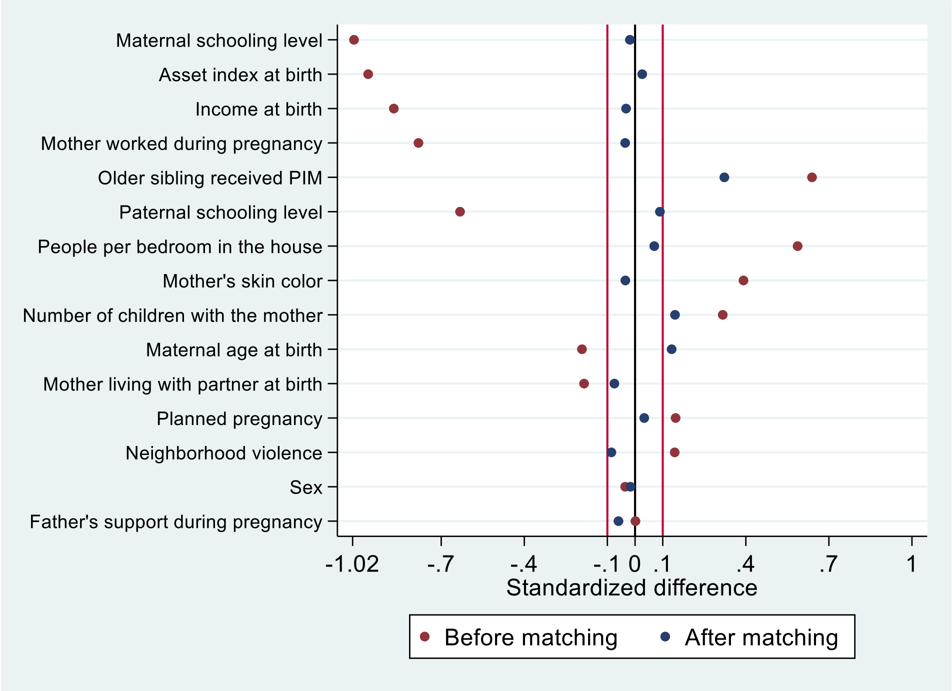

Supplemental Figure 6. Balancing of potential confounders before and after the propensity score matching that was used in the analysis on the effect of Primeira Infância Melhor (PIM) starting during pregnancy (121 pairs).

Supplemental Table 1. Comparison of characteristics between total Pelotas 2015 Birth Cohort sample, analytical sample and individuals excluded from the analyses.

	Whole cohort (N = 4275)	Analytic Sample (N = 3190)	Excluded sample (N = 1085)
	n(%)	n(%)	n(%)
**Sex**			
Female	2111(49.4)	1565(49.1)	546(50.3)
Male	2164(50.6)	1625(50.9)	539(49.7)
Missing data (n)	0	0	0
**Mother's skin color**			
White	3024(70.9)	2297(72.0)	727(67.1)
Black	667(15.6)	489(15.3)	178(16.4)
Brown	551(12.9)	385(12.1)	166(15.3)
Asian	16(0.4)	12(0.4)	4(0.4)
Indigenous	10(0.2)	8(0.3)	2(0.2)
Missing data (n)	7	0	7
**Income at birth (quintiles)**			
Poorest	846(19.8)	573(18.0)	273(25.2)
2nd	859(20.1)	638(20.0)	221(20.4)
3rd	853(20.0)	653(20.5)	200(18.5)
4th	856(20.0)	678(21.3)	178(16.5)
Richest	859(20.1)	649(20.3)	210(19.4)
Missing data (n)	2	0	2
**Asset index at birth (quintiles)**			
Poorest	826(20.0)	597(18.7)	229(24.5)
2nd	825(20.0)	632(19.8)	193(20.6)
3rd	826(20.0)	654(20.5)	172(18.4)
4th	825(20.0)	665(20.8)	160(17.1)
Richest	825(20.0)	643(20.2)	182(19.4)
Missing data (n)	148	0	148
**Maternal schooling level at birth (years)**			
0-4	391(9.2)	238(7.5)	153(14.1)
5-8	1095(25.6)	773(24.2)	322(29.7)
9-11	1458(34.1)	1140(35.7)	318(29.4)
12+	1330(31.1)	1040(32.6)	290(26.8)
Missing data (n)	1	0	1
**Paternal schooling level at birth (years)**			
0-4	537(13.4)	400(12.5)	137(16.5)
5-8	1164(28.9)	941(29.5)	223(26.8)
9-11	1279(31.8)	1041(32.6)	238(28.6)
12+	1043(25.9)	809(25.4)	234(28.1)
Missing data (n)	252	0	252
**People per bedroom in the house at birth**			
2	1680(40.9)	1299(40.7)	381(41.7)
> 2 to 3	1757(42.8)	1398(43.8)	359(39.3)
> 3 to 4	482(11.7)	363(11.4)	119(13.0)
> 4	186(4.5)	131(4.1)	55(6.0)
Missing data (n)	170	0	170
**Number of children living with the mother at birth**			
0	2174(50.9)	1622(50.8)	552(51.0)
1	1341(31.4)	1042(32.7)	299(27.6)
2	453(10.6)	335(10.5)	118(10.9)
3	169(4.0)	115(3.60)	54(5.0)

4 or more	137(3.2)	77(2.4)	60(5.5)
Missing data (n)	1	0	1
**Number of prenatal visits**			
0-5	586(14.0)	389(12.2)	197(19.8)
≥6	3600(86.0)	2802(87.8)	798(80.2)
Missing data (n)	89	0	89
**Maternal age at birth (years)**			
<20	623(14.6)	424(13.3)	199(18.4)
20+	3651(85.4)	2767(86.7)	884(81.6)
Missing data (n)	1	0	1
**Mother living with husband or partner at birth**			
No	607(14.2)	337(10.6)	270(24.9)
Yes	3667(85.8)	2854(89.4)	813(75.1)
Missing data (n)	1	0	1
**Mother worked during pregnancy**			
No	1895(44.3)	1332(41.7)	563(52.0)
Yes	2379(55.7)	1859(58.3)	520(48.0)
Missing data (n)	1	0	1
**Father's support level during pregnancy**			
Little support	462(11.0)	294(9.2)	168(16.7)
Much support	3736(89.0)	2897(90.8)	839(83.3)
Missing data (n)	77	0	77
**Planned pregnancy**			
Yes	2058(48.2)	1625(50.9)	433(40.0)
No	2216(51.9)	1566(49.1)	650(60.0)
Missing data (n)	1	0	1
**Smoking during pregnancy**			
No	3640(85.2)	2777(87.0)	863(79.9)
Yes	631(14.8)	414(13.0)	217(20.1)
Missing data (n)	4	0	4
**Alcohol use during pregnancy**			
No	3957(92.6)	2973(93.2)	984(91.0)
Yes	315(7.4)	218(6.8)	97(9.0)
Missing data (n)	3	0	3
**Maternal arterial hypertension during pregnancy**			
No	3183(74.5)	2371(74.3)	812(75.1)
Yes	1089(25.5)	819(25.7)	270(25.0)
Missing data (n)	3	0	3
**Maternal diabetes mellitus during pregnancy**			
No	3906(91.4)	2908(91.1)	998(92.3)
Yes	366(8.6)	283(8.9)	83(7.7)
Missing data (n)	3	0	3
**Gestational age at birth (weeks)**			
≤ 36	663(15.5)	428(13.4)	235(21.7)
≥ 37	3612(84.5)	2763(86.6)	849(78.3)
Missing data (n)	0	0	0
**Birth weight (grams)**			
≥ 2500	3826(89.9)	2913(91.3)	913(85.6)
< 2500	432(10.2)	278(8.7)	154(14.4)
Missing data (n)	17	0	17
**Apgar 5th minute**			
≥ 7	4221(98.7)	3164(99.2)	1057(97.5)

< 7	54(1.3)	27(0.9)	27(2.5)
Missing data (n)	0	0	0
**Couple's relationship quality**			
Little criticism	2231(64.8)	1827(65.5)	404(62.0)
Medium criticism	711(20.7)	568(20.4)	143(21.9)
Lots of criticism	501(14.6)	396(14.2)	105(16.1)
Missing data (n)	832	400	432
**Depressive maternal symptoms (3 months)**			
No	3264(79.7)	2562(80.3)	702(77.7)
Yes	831(20.3)	629(19.7)	202(22.4)
Missing data (n)	180	0	180
**Main caregiver until 3 months of age**			
Another person	168(4.1)	97(3.0)	71(7.7)
Mother	3941(95.9)	3094(97.0)	847(92.3)
Missing data (n)	166	0	166
**Childcare attendance up to age 2 years**			
Never	2781(67.5)	2109(66.1)	672(72.1)
Sometimes	937(22.7)	732(22.9)	205(22.0)
Always	405(9.8)	350(11.0)	55(5.9)
Missing data (n)	152	0	152
**Neighborhood violence**			
Low	2320(58.3)	1858(58.2)	462(58.3)
Medium	1343(33.7)	1078(33.8)	265(33.5)
High	320(8.0)	255(8.0)	65(8.2)
Missing data (n)	292	0	292
**Any enrollment in PIM up to age 4**			
No	3454(81.3)	2589(81.2)	865(81.5)
Yes	797(18.6)	601(18.8)	196(18.5)
Missing data (n)	24	0	24
**PIM starting during pregnancy**			
Not received PIM	3454(81.3)	2589(81.2)	865(81.5)
Started after birth	640(15.1)	480(15.0)	160(15.1)
Started during pregnancy	157(3.7)	121(3.8)	36(3.4)
Missing data (n)	24	0	24
**Age of entry into PIM (months)**			
Not received PIM	3454(81.3)	2589(81.2)	865(81.5)
Before 6	325(7.7)	253(7.9)	72(6.8)
6 to 12	87(2.1)	65(2.0)	22(2.1)
13 to 24	258(6.1)	196(6.1)	62(5.9)
After 24	127(3.0)	87(2.7)	40(3.8)
Missing data (n)	24	0	24
**Time receiving PIM (months)**			
Not received PIM	3454(81.3)	2589(81.2)	865(81.5)
< 6	54(1.3)	37(1.2)	17(1.6)
6 to 11	293(6.9)	210(6.6)	83(7.8)
12 to 17	198(4.7)	154(4.8)	44(4.1)
18 to 23	136(3.2)	109(3.4)	27(2.5)
24+	116(2.7)	91(2.9)	25(2.4)
Missing data (n)	24	0	24
**Reason for withdrawal from PIM**			
Not received PIM	3454(81.3)	2589(81.2)	865(81.5)
Lack of visitor	270(6.4)	206(6.5)	64(6.0)

Family chose to leave	197(4.6)	149(4.7)	48(4.5)
Daycare admission	113(2.7)	90(2.8)	23(2.2)
Change of neighborhood or municipality	90(2.1)	63(2.0)	27(2.5)
Age limit	23(0.5)	15(0.5)	8(0.8)
Unknown	104(2.5)	78(2.4)	26(2.5)
Missing data (n)	24	0	24
**Number of PIM visitors who accompanied the child**			
Not received PIM	3454(81.3)	2589(81.2)	865(81.5)
1	463(10.9)	339(10.6)	124(11.7)
2	202(4.8)	155(4.9)	47(4.4)
3	92(2.2)	76(2.4)	16(1.5)
4+	40(0.9)	31(1.0)	9(0.8)
Missing data (n)	24	0	24
**Older sibling received PIM**			
No	3962(92.7)	2953(92.6)	1009(93.0)
Yes	313(7.3)	237(7.4)	76(7.0)
Missing data (n)	0	0	0
**Below the 10th percentile on Battelle score at age 4 years**			
No	3234(89.7)	2881(90.3)	353(85.5)
Yes	370(10.3)	310(9.7)	60(14.5)
Missing data (n)	671	0	671
		**Mean(SD)**	
**Battelle total score at age 4 years**	113.4(8.8)	113.7(8.6)	111.2(9.7)
Missing data (n)	671	0	671

Supplemental Table 2. Prevalence of analytical sample characteristics according to whether or not children received Primeira Infância Melhor (PIM).

	Any enrollment in PIM		PIM starting during pregnancy	
	No (N=2589)	Yes (N=601)	No (N=2589)	Yes (N=121)
	N (%)	N (%)	N (%)	N (%)
**Neighborhood violence**		*p<0,001*		*p=0,045*
Low	1575 (60.8)	282 (46.9)	1575 (60.8)	66 (54.6)
Medium	841 (32.5)	237 (39.4)	841 (32.5)	40 (33.1)
High	173 (6.7)	82 (13.6)	173 (6.7)	15 (12.4)
**Mother's skin color**		*p<0,001*		*p<0,001*
White	1965 (75.9)	331 (55.1)	1965 (75.9)	66 (54.6)
Black	334 (12.9)	155 (25.8)	334 (12.9)	29 (24.0)
Brown	275 (10.6)	110 (18.3)	275 (10.6)	25 (20.7)
Asian	11 (0.4)	1 (0.2)	11 (0.4)	0 (0.0)
Indigenous	4 (0.2)	4 (0.7)	4 (0.2)	1 (0.8)
**Maternal schooling level at birth (years)**		*p<0,001*		*p<0,001*
0-4	136 (5.3)	102 (17.0)	136 (5.3)	27 (22.3)
5-8	552 (21.3)	220 (36.6)	552 (21.3)	46 (38.0)
9-11	915 (35.3)	225 (37.4)	915 (35.3)	38 (31.4)
12+	968 (38.1)	54 (9.0)	968 (38.1)	10 (8.3)
**Paternal schooling level at birth (years)**		*p<0,001*		*p<0,001*
0-4	258 (10.0)	142 (23.6)	258 (10.0)	25 (20.7)
5-8	686 (26.5)	255 (42.4)	686 (26.5)	50 (41.3)
9-11	871 (33.6)	170 (28.3)	871 (33.6)	40 (33.1)
12+	774 (29.9)	34 (5.7)	774 (29.9)	6 (5.0)
**Income at birth (quintiles)**		*p<0,001*		*p<0,001*
Poorest	371 (14.3)	202 (33.6)	371 (14.3)	44 (36.4)
2nd	467 (18.0)	171 (28.5)	467 (18.0)	38 (31.4)
3rd	521 (20.1)	132 (22.0)	521 (20.1)	20 (16.5)
4th	606 (23.4)	71 (11.8)	606 (23.4)	16 (13.2)
Richest	624 (24.1)	25 (4.2)	624 (24.1)	3 (2.5)
**Asset index at birth (quintiles)**		*p<0,001*		*p=0,001*
Poorest	383 (14.8)	214 (35.6)	383 (14.8)	45 (37.2)
2nd	459 (17.7)	173 (28.8)	459 (17.7)	40 (33.1)
3rd	528 (20.4)	126 (21.0)	528 (20.4)	22 (18.2)
4th	595 (23.0)	69 (11.5)	595 (23.0)	9 (7.4)
Richest	624 (24.1)	19 (3.2)	624 (24.1)	5 (4.1)
**People per bedroom in the house at birth**		*p<0,001*		*p<0,001*
2	1133 (43.8)	165 (27.5)	1133 (43.8)	29 (24.0)
> 2 to 3	1126 (43.5)	272 (45.3)	1126 (43.5)	54 (44.6)
> 3 to 4	254 (9.8)	109 (18.1)	254 (9.8)	25 (20.7)
> 4	76 (2.9)	55 (9.2)	76 (2.9)	13 (10.7)
**Number of prenatal visits**		*p<0,001*		*p=0,815*
0-5	282 (10.9)	106 (17.6)	282 (10.9)	14 (11.6)
≥ 6	2307 (89.1)	495 (82.4)	2307 (89.1)	107 (88.4)
**Maternal age at birth (years)**		*p<0,001*		*p=0,003*
< 20	302 (11.7)	122 (20.3)	302 (11.7)	25 (20.7)
20+	2287 (88.3)	479 (79.7)	2287 (88.3)	96 (79.3)
**Number of children living with the mother at birth**		*p<0,001*		*p=0,003*
0	1372 (53.0)	249 (41.4)	1372 (53.0)	45 (37.2)

1	843 (32.6)	199 (31.1)	843 (32.6)	48 (39.7)
2	247 (9.5)	88 (14.6)	247 (9.5)	17 (14.1)
3	82 (3.2)	33 (5.5)	82 (3.2)	5 (4.1)
4 or more	45 (1.7)	32 (5.3)	45 (1.7)	6 (5.0)
**Mother living with husband or partner at birth**		*p=0,001*		*p=0,03*
No	250 (9.7)	87 (14.5)	250 (9.7)	19 (15.7)
Yes	2339 (90.3)	514 (85.5)	2339 (90.3)	102 (84.3)
**Mother worked during pregnancy**		*p<0,001*		*p<0,001*
No	967 (37.4)	364 (60.6)	967 (37.4)	89 (73.6)
Yes	1622 (62.7)	237 (39.4)	1622 (62.7)	32 (26.5)
**Father's support during pregnancy**		*p=0.133*		*p=0.826*
Little support	229 (8.9)	65 (10.8)	229 (8.9)	10 (8.3)
Much support	2360 (91.2)	536 (89.2)	2360 (91.2)	111 (91.7)
**Couple's relationship quality**		*p<0,001*		*p=0.392*
Little criticism	1541 (67.1)	285 (57.7)	1541 (67.1)	62 (63.3)
Medium criticism	436 (19.0)	132 (26.7)	436 (19.0)	24 (24.5)
Lots of criticism	319 (13.9)	77 (15.6)	319 (13.9)	12 (12.2)
**Planned pregnancy**		*p=0,001*		*p=0.028*
Yes	1355 (52.3)	270 (44.9)	1355 (52.3)	51 (42.2)
No	1234 (47.7)	331 (55.1)	1234 (47.7)	70 (57.9)
**Smoking during pregnancy**		*p<0,001*		*p=0,002*
No	2293 (88.6)	483 (80.4)	2293 (88.6)	96 (79.3)
Yes	296 (11.4)	118 (19.6)	296 (11.4)	25 (20.7)
**Alcohol use during pregnancy**		*p=0.848*		*p=0.642*
No	2411 (93.1)	561 (93.3)	2411 (93.1)	114 (94.2)
Yes	178 (6.9)	40 (6.7)	178 (6.9)	7 (5.8)
**Maternal arterial hypertension during pregnancy**		*p=0.860*		*p=0.841*
No	1926 (74.4)	445 (74.0)	1926 (74.4)	91 (75.2)
Yes	663 (25.6)	156 (26.0)	663 (25.6)	30 (24.8)
**Maternal diabetes mellitus during pregnancy**		*p=0.669*		*p=0.455*
No	2362 (91.2)	545 (90.7)	2362 (91.2)	108 (89.3)
Yes	227 (8.8)	56 (9.3)	227 (8.8)	13 (10.7)
**Depressive maternal symptoms (3 months)**		*p<0,001*		*p<0,314*
No	2126 (82.1)	436 (72.6)	2126 (82.1)	95 (78.5)
Yes	463 (17.9)	165 (27.5)	463 (17.9)	26 (21.5)
**Gestational age at birth (weeks)**		*p=0.476*		*p=0.188*
≥ 37	2247 (86.8)	515 (85.7)	2247 (86.8)	110 (90.9)
≤ 36	342 (13.2)	86 (14.3)	342 (13.2)	11 (9.1)
**Birth weight (grams)**		*p=0.458*		*p=0.457*
≥ 2500	2368 (91.5)	544 (90.5)	2368 (91.5)	113 (93.4)
< 2500	221 (8.5)	57 (9.5)	221 (8.5)	8 (6.6)
**Apgar 5th minute**		*p=0.652*		*p=0.985*
≥ 7	2568 (99.2)	595 (99.0)	2568 (99.2)	120 (99.2)
< 7	21 (0.8)	6 (1.0)	21 (0.8)	1 (0.8)
**Sex**		*p=0.506*		*p=0.725*
Female	1262 (48.7)	302 (50.3)	1262 (48.7)	57 (47.1)
Male	1327 (51.3)	299 (49.8)	1327 (51.3)	64 (52.9)
**Main caregiver until 3 months of age**		*p=0,002*		*p=0,302*
Another person	67 (2.6)	30 (5.0)	67 (2.6)	5 (4.1)
Mother	2522 (97.4)	571 (95.0)	2522 (97.4)	116 (95.9)
**Childcare attendance up to age 2 years**		*p<0,001*		*p=0,004*
Never	1631 (63.0)	477 (79.4)	1631 (63.0)	93 (76.86)

Sometimes	628 (24.3)	104 (17.3)	628 (24.3)	22 (18.2)
Always	330 (12.8)	20 (3.3)	330 (12.8)	6 (5.0)
**Below the 10th percentile on Battelle score at age 4 years**		*p=0,017*		*p=0,529*
No	2353 (90.9)	527 (87.7)	2353 (90.9)	112 (92.6)
Yes	236 (9.1)	74 (12.3)	236 (9.1)	9 (7.4)

Supplemental Table 3. Balancing of potential confounders before and after the propensity score matching that was used in the analysis on the effect of any enrollment in Primeira Infância Melhor (PIM) (601 pairs).

Confounder	Mean in treated	Mean in untreated	Standardized difference	
			After matching	Before matching
Neighborhood violence	0.67	0.65	0.023	0.327
Mother's skin color (others/white)	0.42	0.44	-0.034	0.436
Maternal schooling level (years)	7.98	8.06	-0.023	-0.857
Paternal schooling level	1.13	1.18	-0.047	-0.802
Income at birth (quintiles)	2.28	2.35	-0.054	-0.830
Asset index at birth (quintiles)	2.20	2.21	-0.011	-0.872
People per bedroom in the house	1.14	1.10	0.054	0.486
Number of prenatal visits (> 5)	0.84	0.84	0.002	-0.199
Maternal age at birth (> 19 years)	0.83	0.81	0.072	-0.208
Number of children living with the mother	1.03	0.94	0.092	0.356
Mother living with partner at birth	0.97	0.96	0.073	-0.045
Mother worked during pregnancy	0.39	0.42	-0.054	-0.505
Father's support during pregnancy	0.95	0.94	0.022	-0.006
Couple's relationship quality	0.58	0.54	0.053	0.151
Planned pregnancy	0.51	0.50	0.012	0.132
Smoking during pregnancy	0.19	0.20	-0.023	0.241
Alcohol use during pregnancy	0.06	0.06	-0.029	-0.015
Maternal hypertension during pregnancy	0.27	0.29	-0.042	0.029
Maternal diabetes during pregnancy	0.11	0.09	0.054	0.049
Depressive maternal symptoms (3 months)	0.25	0.27	-0.056	0.209
Gestational age (< 37 weeks)	0.15	0.13	0.032	0.057
Birth weight (grams)	3161.36	3151.46	0.018	-0.090
Apgar 5th minute	0.01	0.01	0.002	0.043
Sex	1.51	1.50	0.024	0.050
Main caregiver until 3 months	0.96	0.98	-0.089	-0.131
Childcare attendance up to age 2 years	0.22	0.24	-0.019	-0.442
Older sibling received PIM	0.19	0.17	0.043	0.435

Supplemental Table 4. Balancing of confounders between two randomly generated control groups used to test interaction with starting Primeira Infância Melhor (PIM) during pregnancy or after birth. N group 1 = 542; N group 2 = 2054.

Potential confounder	Mean in group 1	Mean in group 2	Standardized difference
Neighborhood violence	0.44	0.47	-0.044
Mother's skin color (others/white)	0.22	0.24	-0.046
Maternal schooling level (years)	11.01	11.11	-0.026
Paternal schooling level	1.86	1.87	-0.011
Income at birth (quintiles)	3.31	3.35	-0.031
Asset index at birth (quintiles)	3.27	3.39	-0.084
People per bedroom in the house	0.75	0.71	0.055
Number of prenatal visits (> 5)	0.90	0.92	-0.041
Maternal age at birth (> 19 years)	0.13	0.12	0.071
Number of children living with the mother	0.68	0.68	-0.005
Mother living with partner at birth	0.98	0.99	-0.081
Mother worked during pregnancy	0.63	0.64	-0.006
Father's support during pregnancy	0.95	0.96	-0.053
Couple's relationship quality	0.46	0.49	-0.045
Planned pregnancy	0.43	0.47	-0.071
Smoking during pregnancy	0.11	0.10	0.015
Alcohol use during pregnancy	0.06	0.05	0.050
Maternal hypertension during pregnancy	0.26	0.27	-0.031
Maternal diabetes during pregnancy	0.09	0.10	-0.048
Depressive maternal symptoms (3 months)	0.17	0.17	0.004
Gestational age (< 37 weeks)	0.13	0.12	0.038
Birth weight (grams)	3203.09	3.238.65	-0.067
Apgar 5th minute	0.01	0.01	0.022
Sex	1.49	1.48	0.026
Main caregiver until 3 months	0.98	0.99	-0.021
Childcare attendance up to age 2 years	0.49	0.51	-0.023
Older sibling received PIM	0.05	0.03	0.095

Supplemental Table 5. Balancing of confounders before and after propensity score matching used in the analysis of effect of the Primeira Infância Melhor (PIM) starting after birth (480 pairs).

Potential confounder	Mean in treated	Mean in untreated	Standardized difference	
			After matching	Before matching
Neighborhood violence	0.69	0.69	0.004	0.380
Mother's skin color (others/white)	0.43	0.42	0.014	0.453
Maternal schooling level (years)	8.22	8.35	-0.037	-0.786
Paternal schooling level	1.13	1.16	-0.036	-0.803
Income at birth (quintiles)	2.30	2.36	-0.053	-0.807
Asset index at birth (quintiles)	2.23	2.25	-0.012	-0.829
People per bedroom in the house	1.12	1.10	0.022	0.444
Number of prenatal visits (> 5)	0.83	0.83	-0.016	-0.229
Maternal age at birth (> 19 years)	0.83	0.83	-0.001	-0.214
Number of children living with the mother	1.03	1.00	0.030	0.349
Mother living with partner at birth	0.97	0.96	0.090	-0.011
Mother worked during pregnancy	0.43	0.43	0.003	-0.421
Father's support during pregnancy	0.94	0.93	0.057	-0.008
Couple's relationship quality	0.60	0.53	0.098	0.189
Planned pregnancy	0.51	0.51	-0.017	0.144
Smoking during pregnancy	0.18	0.20	-0.047	0.209
Alcohol use during pregnancy	0.06	0.06	-0.013	-0.019
Maternal hypertension during pregnancy	0.27	0.31	-0.080	0.034
Maternal diabetes during pregnancy	0.10	0.07	0.095	0.045
Depressive maternal symptoms (3 months)	0.26	0.27	-0.010	0.235
Gestational age (< 37 weeks)	0.16	0.15	0.028	0.093
Birth weight (grams)	3129.40	3147.90	-0.034	-0.135
Apgar 5th minute	0.01	0.01	0.004	0.043
Sex	1.53	1.52	0.019	0.071
Main caregiver until 3 months	0.96	0.98	-0.097	-0.124
Childcare attendance up to age 2 years	0.21	0.20	0.019	-0.457
Older sibling received PIM	0.17	0.18	-0.038	0.382

Viegas da Silva E, *et al. BMJ Global Health* 2022; 7:e007116. doi: 10.1136/bmjgh-2021-007116

Supplemental Table 6. Balancing of confounders before and after propensity score matching used in the analysis of effect of the Primeira Infância Melhor (PIM) starting during pregnancy (121 pairs).

Potential confounder	Mean in treated	Mean in untreated	Standardized difference	
			After matching	Before matching
Neighborhood violence	0.58	0.64	-0.085	0.143
Mother's skin color (others/white)	0.45	0.47	-0.035	0.392
Maternal schooling level (years)	7.33	7.40	-0.019	-1.015
Paternal schooling level	1.22	1.14	0.090	-0.632
Income at birth (quintiles)	2.14	2.18	-0.032	-0.871
Asset index at birth (quintiles)	2.08	2.05	0.026	-0.964
People per bedroom in the house	1.18	1.12	0.069	0.587
Maternal age at birth (> 19 years)	0.79	0.74	0.132	-0.192
Number of children living with the mother	1.00	0.86	0.144	0.317
Mother living with partner at birth	0.84	0.87	-0.075	-0.184
Mother worked during pregnancy	0.26	0.28	-0.036	-0.782
Father's support during pregnancy	0.92	0.93	-0.060	0.001
Planned pregnancy	0.58	0.56	-0.033	0.146
Sex	1.47	1.48	-0.017	-0.036
Older sibling received PIM	0.25	0.14	0.323	0.639

Supplemental Table 7. Effect modification of the Primeira Infância Melhor (PIM) according to starting during pregnancy (121 pairs) or after birth (480 pairs), in relation to standardized development scores and prevalence of belonging to the group below the 10th percentile of whole cohort.

**Outcome**	**Unadjusted[a]**				**Matched[b]**				**Matched with double adjustment[c]**				Heterogeneity p-value[d]
	Started during pregnancy N = 663		Started after Birth N = 2534		Started during pregnancy N = 242		Started after Birth N = 960		Started during pregnancy N = 242		Started after Birth N = 960		
	β	95% CI	β	95% CI	β	95% CI	β	95% CI	β	95% CI	β	95% CI	
Linear regression for mean BDI score													
Total BDI score (SD)	-0.07	-0.27 to 0.14	-0.22	-0.32 to -0.13	0.17	-0.06 to 0.41	-0.06	-0.18 to 0.07	0.19	-0.02 to 0.40	-0.03	-0.15 to 0.10	0.080
Personal-social (SD)	-0.05	-0.25 to 0.14	-0.19	-0.28 to -0.10	0.08	-0.17 to 0.33	-0.03	-0.15 to 009	0.06	-0.17 to 0.29	0.00	-0.12 to 0.12	0.669
Adaptive (SD)	0.05	-0.15 to 0.24	0.08	-0.01 to 0.17	0.02	-0.20 to 0.23	0.06	-0.04 to 0.17	0.05	-0.14 to 0.24	0.07	-0.03 to 0.18	0.849
Motor (SD)	0.01	-0.19 to 0.21	-0.09	-0.19 to 0.01	0.18	-0.07 to 0.43	-0.03	-0.16 to 0.10	0.18	-0.07 to 0.42	-0.01	-0.13 to 0.12	0.187
Communication (SD)	-0.10	-0.30 to 0.11	-0.21	-0.31 to -0.12	0.16	-0.06 to 0.39	-0.03	-0.15 to 0.08	0.19	-0.02 to 0.39	-0.02	-0.13 to 0.10	0.092
Cognitive (SD)	-0.12	-0.31 to 0.08	-0.32	-0.42 to -0.23	0.16	-0.04 to 0.37	-0.15	-0.28 to -0.01	0.19	-0.00 to 0.39	-0.12	-0.25 to 0.01	0.008
Poisson regression for belonging to the group below the 10th percentile													
	PR	95% CI	PR	95% CI	PR	95% CI	PR	95% CI	PR	95% CI	PR	95% CI	
Low development score	0.76	0.39 to 1.50	1.50	1.15 to 1.96	0.45	0.20 to 1.00	1.14	0.82 to 1.60	0.40	0.18 to 0.89	1.12	0.81 to 1.54	0.020

PR = prevalence ratio; CI = confidence interval

[a]Comparison between intervention group and possible controls group without adjustment.

[b]Paired analysis comparing the intervention group with the matched control group.

[c]Paired analysis comparing the intervention group with the matched control group with double adjustment for confounders included in the propensity score prediction for starting during pregnancy (neighborhood violence, mother's skin color (others/white), maternal age at birth (> 19 years), sex, maternal schooling level (years), paternal schooling level, income at birth (quintiles), asset index at birth (quintiles), people per bedroom in house, number of children with the mother, mother living with partner, mother worked during pregnancy, father's support during pregnancy, planned pregnancy, older sibling received the PIM) and for starting after birth (neighborhood violence, mother's skin color (others/white), maternal age at birth (> 19 years), sex, maternal schooling level (years), paternal schooling level, income at birth (quintiles), asset index at birth (quintiles), number of prenatal visits (> 5), people per bedroom in house, number of children with the mother, mother living with partner, mother worked during pregnancy, father's support during pregnancy, planned pregnancy, depressive maternal symptoms (3 months), birth weight (grams), smoking during pregnancy, alcohol use during pregnancy, maternal hypertension during pregnancy, maternal diabetes during pregnancy, gestational age (< 37 weeks), main caregiver until 3 months, childcare attendance up to age 2 years, older sibling received the PIM).

[d]Cochran's Q heterogeneity chi-square test

Supplemental Table 8. Association between early childhood development at age 4 years and analytical sample characteristics (N=3190).

	Standardized BDI Total score		Below the 10th percentile on BDI	
			No	Yes
	β	p-valor	N (%)	N (%)
**Neighborhood violence**				*p=0.027*
Low	0		1698 (91.4)	159 (8.6)
Medium	-0.12	*0.001*	953 (88.4)	125 (11.6)
High	-0.04	0.576	229 (89.8)	26 (10.2)
**Mother's skin color**				*p=0.007*
White	0		2099 (91.4)	197 (8.6)
Black	-0.22	*<0.001*	430 (87.9)	59 (12.1)
Brown	-0.20	*<0.001*	334 (86.8)	51 (13.3)
Asian	-0.27	*0.345*	11 (91.7)	1 (8.3)
Indigenous	-0.71	*0.039*	6 (75.0)	2 (25.0)
**Maternal schooling level at birth (years)**				*p<0.001*
0-4	0		203 (85.3)	35 (14.7)
5-8	0.14	*0.05*	663 (85.9)	109 (14.1)
9-11	0.34	*<0.001*	1043 (91.5)	97 (8.5)
12+	0.55	*<0.001*	971 (93.4)	69 (6.6)
**Paternal schooling level at birth (years)**				*p<0.001*
0-4	0		341 (85.3)	59 (14.8)
5-8	0.22	*<0.001*	832 (88.4)	109 (11.6)
9-11	0.33	*<0.001*	946 (90.9)	95 (9.1)
12+	0.58	*<0.001*	761 (94.2)	47 (5.8)
**Income at birth (quintiles)**				*p<0.001*
Poorest	0		490 (85.5)	83 (14.5)
2nd	0.09	0.125	558 (87.5)	80 (12.5)
3rd	0.26	*<0.001*	596 (91.3)	57 (8.7)
4th	0.31	*<0.001*	619 (91.4)	58 (8.6)
Richest	0.54	*<0.001*	617 (95.1)	32 (4.9)
**Asset index at birth (quintiles)**				*p<0.001*
Poorest	0		519 (86.9)	78 (13.1)
2nd	0.11	*0.041*	557 (88.1)	75 (11.9)
3rd	0.21	*<0.001*	584 (89.3)	70 (10.7)
4th	0.30	*<0.001*	612 (92.2)	52 (7.8)
Richest	0.50	*<0.001*	608 (94.6)	35 (5.4)
**People per bedroom in the house at birth**				*p=0.132*
2	0		1180 (90.9)	118 (9.1)
> 2 to 3	-0.04	*0.252*	1267 (90.6)	131 (9.4)

> 3 to 4	-0.17	*0.003*	321 (88.4)	42 (11.6)
> 4	-0.26	*0.003*	112 (85.5)	19 (14.5)
**Number of prenatal visits**				*p=0.002*
0-5	0		333 (85.8)	55 (14.2)
≥ 6	0.31	*<0.001*	2547 (90.9)	255 (9.1)
**Maternal age at birth (years)**				*p=0.231*
< 20	0		376 (88.7)	48 (11.3)
20+	0.19	*<0.001*	2504 (90.5)	262 (9.7)
**Number of children living with the mother at birth**				*p=0.722*
0	0		1472 (90.8)	149 (9.2)
1	-0.05	*0.173*	936 (89.8)	106 (10.2)
2	-0.11	*0.06*	300 (89.6)	35 (10.5)
3	-0.20	*0.037*	101 (87.8)	14 (12.2)
4 or more	-0.20	*0.076*	71 (92.2)	6 (7.8)
**Mother living with husband or partner at birth**				*p=0.466*
No	0		308 (91.4)	29 (8.6)
Yes	0.10	*<0.092*	2572 (90.2)	281 (9.9)
**Mother worked during pregnancy**				*p=0.002*
No	0		1176 (88.4)	155 (11.7)
Yes	0.241	*<0.001*	1704 (91.7)	155 (8.3)
**Father's support during pregnancy**				*p=0.262*
Little support	0		260 (88.4)	34 (11.6)
Much support	0.15	*0.012*	2620 (90.5)	276 (9.5)
**Couple's relationship quality**				*p=0.325*
Little criticism	0		1661 (91.0)	165 (9.0)
Medium criticism	-0.08	*0.103*	506 (89.1)	62 (10.9)
Lots of criticism	-0.08	*0.145*	354 (89.4)	42 (10.6)
**Planned pregnancy**				*p=0.192*
Yes	0		1478 (91.0)	147 (9.1)
No	-0.11	*0.001*	1402 (89.6)	163 (10.4)
**Smoking during pregnancy**				*p=0.023*
No	0		2519 (90.7)	257 (9.3)
Yes	-0.20	*<0.001*	361 (87.2)	53 (12.8)
**Alcohol use during pregnancy**				*p=0.605*
No	0		2681 (90.2)	291 (9.8)
Yes	-0.09	*0.211*	199 (91.3)	19 (8.7)
**Maternal arterial hypertension during pregnancy**				*p=0.089*
No	0		2153 (90.8)	218 (9.2)
Yes	-0.10	*0.017*	727 (88.8)	92 (11.2)
**Maternal diabetes mellitus during pregnancy**				*p=0.046*
No	0		2634 (90.6)	273 (9.4)

Yes	-0.13	*0.033*	246 (86.9)	37 (13.1)
**Depressive maternal symptoms (3 months)**				*p=0.134*
No	0		2323 (90.7)	239 (9.3)
Yes	-0.09	*0.037*	557 (88.7)	71 (11.3)
**Gestational age at birth (weeks)**				*p=0.019*
≥ 37	0		2507 (90.8)	255 (9.2)
≤ 36	-0.17	*0.001*	373 (87.2)	55 (12.9)
**Birth weight (grams)**				*p=0.003*
≥ 2500	0		2643 (90.8)	269 (9.2)
< 2500	-0.19	*0.002*	237 (85.3)	41 (14.8)
**Apgar 5th minute**				*p=0.369*
≥ 7	0		2857 (90.3)	306 (9.7)
< 7	-0.36	*0.055*	23 (85.2)	4 (14.8)
**Sex**				*p<0.001*
Male	0		1396 (85.9)	230 (14.2)
Female	0.39	*<0.001*	1484 (94.9)	80 (5.1)
**Main caregiver until 3 months of age**				*p=0.370*
Another person	0		85 (87.6)	12 (12.4)
Mother	0.17	*0.085*	2795 (90.4)	298 (9.6)
**Childcare attendance up to age 2 years**				*p=0.001*
Never	0		1875 (89.0)	233 (11.1)
Sometimes	0.15	*<0.001*	675 (92.2)	57 (7.8)
Always	0.25	*<0.001*	330 (94.3)	20 (5.7)
**Age of entry into PIM (months)**				*p=0.198*
Before 6	0		227 (89.7)	26 (10.3)
6 to 12	-0.04	*0.785*	60 (92.3)	5 (7.7)
13 to 24	-0.15	*0.095*	165 (84.2)	31 (15.8)
After 24	0.03	*0.826*	75 (86.2)	12 (13.8)
**Time receiving PIM (months)**				*p=0.247*
< 6	0		34 (91.9)	3 (8.1)
6 to 11	-0.15	*0.375*	185 (88.1)	25 (11.9)
12 to 17	-0.07	*0.684*	136 (88.3)	18 (11.7)
18 to 23	-0.33	*0.069*	89 (81.7)	20 (18.4)
24+	-0.11	*0.555*	83 (91.2)	8 (8.8)
**Reason for withdrawal from PIM**				*p=0.656*
Lack of visitor	0		175 (85.0)	31 (15.1)
Family chose to leave	0.13	*0.213*	134 (89.9)	15 (10.1)
Daycare admission	0.11	*0.379*	81 (90.0)	9 (10.0)
Change of neighborhood or municipality	0.11	*0.780*	54 (85.7)	9 (14.3)
Age limit	0.43	*0.091*	14 (93.3)	1 (6.7)
Unknown	-0.04	*0.729*	69 (88.5)	9 (11.5)

**Older sibling received PIM**				*p=0.012*
No	0		2677 (90.7)	276 (9.4)
Yes	-0.25	*<0.001*	203 (85.7)	34 (14.4)

Supplemental Table 9. Primeira Infância Melhor (PIM) characteristics among children who received the intervention (analytic sample).

	Any enrollment in PIM N = 601	Starting during pregnancy N = 121	Starting after birth N = 480
	N(%)	N(%)	N(%)
**Age of entry into PIM (months)**			
Before 6	253(42.1)	121(100.0)	132(27.5)
6 to 12	65(10.8)	-	65(13.5)
13 to 24	196(32.6)	-	196(40.8)
After 24	87(14.5)	-	87(18.1)
**Time receiving PIM (months)**			
< 6	37(6.2)	10(8.3)	27(5.6)
6 to 11	210(34.9)	31(25.6)	179(37.3)
12 to 17	154(25.6)	17(14.1)	137(28.5)
18 to 23	109(18.1)	27(22.3)	82(17.1)
24+	91(15.1)	36(29.8)	55(11.5)
**Reason for withdrawal from PIM**			
Lack of visitor	206(34.3)	43(35.5)	163(33.4)
Family chose to leave	149(24.8)	30(24.8)	119(24.8)
Daycare admission	90(15.0)	19(15.7)	71(14.8)
Change of neighborhood or municipality	63(10.5)	12(9.9)	51(10.6)
Age limit	15(2.5)	3(2.5)	12(2.5)
Unknown	78(13.0)	14(11.6)	64(13.3)
**Number of PIM visitors who accompanied the child**			
1	339(56.4)	37(30.6)	302(62.9)
2	155(25.79)	40(33.1)	115(24.0)
3	76(12.7)	27(22.3)	49(10.2)
4+	31(5.2)	17(14.1)	14(2.9)
**Older sibling received PIM**			
No	486(80.1)	91(75.2)	395(82.3)
Yes	115(19.1)	30(24.8)	85(17.7)

Supplemental Table 10. Number of different visitors who accompanied the child according to the time receiving PIM (analytic sample).

Number of PIM visitors who accompanied the child	Time receiving PIM (months)				
	**< 6**	**6 to 11**	**12 to 17**	**18 to 23**	**24+**
	N(%)	N(%)	N(%)	N(%)	N(%)
1	34(91.9)	185(88.1)	85(55.2)	28(25.7)	7(7.7)
2	3(8.1)	21(10.0)	55(35.7)	51(46.8)	25(27.5)
3	0	4(1.9)	14(9.1)	22(20.2)	36(39.6)
4+	0	0	0	8(7.3)	23(25.3)

Supplemental Table 11. Missing data in analytical sample for potential confounder variables.

Variables	Missing data	
	**n**	**%**
Couple's relationship quality	629	17.50
Paternal schooling level at birth	252	5.89
Asset index at birth	148	3.46
Number of prenatal visits	65	1.80
Depressive maternal symptons (3 months)	63	1.75
Father's support during pregnancy	59	1.64
People per bedroom in the house at birth	57	1.58
Main caregiver until 3 months of age	54	1.50
Neighborhood violence	19	0.53
Childcare attendance uo to age 2 years	17	0.47
Birth weight (grams)	17	0.47
Mother's skin color	6	0.17
Smoking during pregnancy	4	0.11
Maternal arterial hypertension during pregnancy	3	0.09
Maternal diabetes mellitus during pregnancy	3	0.09
Alcohol use during pregnancy	3	0.09
Income at birth (quintiles)	2	0.06
Mother living with husband or partner at birth	1	0.03
Number of children living with the mother at birth	1	0.03
Planned pregnancy	1	0.03
Mother worked during pregnancy	1	0.03
Maternal age at birth (years)	1	0.03
Maternal schooling level at birth (years)	1	0.03
Gestational age	0	0.00
Apgar 5th minute	0	0.00
Sex	0	0.00
Older sibling received PIM	0	0.00

Supplemental Table 12. Unadjusted association between time receiving PIM and early childhood development at age 4 years, among PIM children who started intervention during pregnancy (n = 121) and who started after birth (n = 480).

	**Standardized BDI (mean differences)[a]**	**Below the 10th percentile (prevalence ratios)[b]**
**Started during pregnancy (121 children)**		
**Time receiving PIM (months)**	***p=0.621****	***p=0.403****
<12	0 (Ref.)	1 (Ref.)
12 to 23	-0.09 (-0.48 to 0.31)	1.55 (0.39 to 6.13)
24 or more	0.12 (-0.30 to 0.53)	0.38 (0.04 to 3.52)
**Started after birth (480 children)**		
**Time receiving PIM (months)**	***p=0.840****	***p=0.667****
<12	0 (Ref.)	1 (Ref.)
12 to 23	-0.04 (-0.22 to 0.14)	1.24 (0.77 to 2.01)
24 or more	-0.07 (-0.36 to 0.21)	1.05 (0.48 to 2.30)

[a]Linear regression

[b]Poisson regression

*p-valeu of wald-test

Supplemental Table 13. Exploratory analysis of interaction between enrollment in PIM (separately by subgroups who started intervention during pregnancy or after birth) and time receiving the program predicting early childhood development at age 4 years. Matched analysis with double adjustment comparing with control groups.

	Standardized BDI (mean differences)[a]	Below the 10th percentile (prevalence ratios)[b]
**Started during pregnancy (121 pairs)**		
**PIM started during pregnancy#Time receiving PIM (vs matched controls)**	***p=0.856****	***p=0.150****
In pregnancy#<12	0 (Ref.)	1 (Ref.)
In pregnancy#12 to 23	-0.16 (-0.73 to 0.41)	2.38 (0.40 to 14.20)
In pregnancy#24 or more	0.10 (-0.65 to 0.46)	0.37 (0.04 to 3.39)
**Started after birth (480 pairs)**		
**PIM started after birth#Time receiving PIM (vs matched controls)**	***p=0.861*****	***p=0.228*****
After birth#<12	0 (Ref.)	1 (Ref.)
After birth#12 to 23	-0.07 (-0.34 to 0.20)	1.69 (0.83 to 3.42)
After birth#24 or more	0.00 (-0.42 to 0.42)	0.84 (0.31 to 2.28)

[a]Linear regression

[b]Poisson regression

*p-valeu of adjusted wald-test for interaction between PIM started during pregnancy and time receiving PIM

**p-valeu of adjusted wald-test for interaction between PIM started after birth and time receiving PIM

Paired analysis comparing the intervention group with the matched control group with double adjustment. Note that to estimate the interaction effect, for the control group, the time in PIM was imputed to equal the value of the matched PIM child from the same pair. The confounders included in the propensity score prediction for starting during pregnancy (neighborhood violence, mother's skin color (others/white), maternal age at birth (> 19 years), sex, maternal schooling level (years), paternal schooling level, income at birth (quintiles), asset index at birth (quintiles), people per bedroom in house, number of children with the mother, mother living with partner, mother worked during pregnancy, father's support during pregnancy, planned pregnancy, older sibling received the PIM) and for starting after birth (neighborhood violence, mother's skin color (others/white), maternal age at birth (> 19 years), sex, maternal schooling level (years), paternal schooling level, income at birth (quintiles), asset index at birth (quintiles), number of prenatal visits (> 5), people per bedroom in house, number of children with the mother, mother living with partner, mother worked during pregnancy, father's support during pregnancy, planned pregnancy, depressive maternal symptoms (3 months), birth weight (grams), smoking during pregnancy, alcohol use during pregnancy, maternal hypertension during pregnancy, maternal diabetes during pregnancy, gestational age (< 37 weeks), main caregiver until 3 months, childcare attendance up to age 2 years, older sibling received the PIM).

Supplemental Box 1. Measurements of the covariates considered for propensity score estimates.

Group	Covariates	Follow-up in which it was collected
27 covariates considered to estimate the PS of any enrollment in PIM and the PS of the stratum with initiation of the intervention after birth	Neighborhood violence: low, medium or high	48-month assessment
	Mother's skin color: white/others	Perinatal assessment
	Maternal schooling level: years	Perinatal assessment
	Paternal schooling level: 0 to 4 years, 5 to 8 years, 9 to 11 years, 12 or more	Perinatal assessment
	Income at birth: quintiles	Perinatal assessment
	Asset index at birth: quintiles	Perinatal assessment
	People per bedroom in the house: ≤ 2, 2.1 – 3.0, 3.1 – 4.0 or > 4	Perinatal assessment
	Number of prenatal visits: ≤ 5 or > 5	Perinatal assessment
	Maternal age at birth: < 20 or ≥ 20	Perinatal assessment
	Number of children living with the mother: 0, 1, 2, 3, 4 or more	Perinatal assessment
	Mother living with partner at birth: no or yes	Perinatal assessment
	Mother worked during pregnancy: no or yes	Perinatal assessment
	Father's support during pregnancy: little support or much support	Perinatal assessment
	Couple's relationship quality: little criticism, medium criticism or lots of criticism	3-month assessment
	Planned pregnancy: yes or no	Perinatal assessment
	Smoking during pregnancy: no or yes	Perinatal assessment
	Alcohol use during pregnancy: no or yes	Perinatal assessment
	Maternal hypertension during pregnancy: no or yes	Perinatal assessment
	Maternal diabetes during pregnancy: no or yes	Perinatal assessment
	Depressive maternal symptoms (3 months): no or yes	3-month assessment
	Gestational age: < 37 weeks or ≥ 37 weeks	Perinatal assessment
	Birth weight: grams	Perinatal assessment
	Apgar 5th minute: < 7 or ≥ 7	Perinatal assessment
	Sex: male/female	Perinatal assessment
	Main caregiver until 3 months: another person or mother	3-month assessment
	Childcare attendance up to age 2 years: never, sometimes or always	24-month assessment
	Older sibling received PIM: no or yes	Link with PIM database (*Sep*/2020)
15 covariates considered to estimate	Neighborhood violence: low, medium or high	48-month assessment
	Mother's skin color: white/others	Perinatal assessment

the PS of the stratum with initiation of the intervention during pregnancy	Maternal schooling level: years	Perinatal assessment
	Paternal schooling level: 0 to 4 years, 5 to 8 years, 9 to 11 years, 12 or more	Perinatal assessment
	Income at birth: quintiles	Perinatal assessment
	Asset index at birth: quintiles	Perinatal assessment
	People per bedroom in the house: ≤ 2, 2.1 – 3.0, 3.1 – 4.0 or > 4	Perinatal assessment
	Maternal age at birth: < 20 or ≥ 20	Perinatal assessment
	Number of children living with the mother: 0, 1, 2, 3, 4 or more	Perinatal assessment
	Mother living with partner at birth: no or yes	Perinatal assessment
	Mother worked during pregnancy: no or yes	Perinatal assessment
	Father's support during pregnancy: little support or much support	Perinatal assessment
	Planned pregnancy: yes or no	Perinatal assessment
	Sex: male/female	Perinatal assessment
	Older sibling received PIM: no or yes	Link with PIM database (*Sep*/2020)

Viegas da Silva E, *et al. BMJ Global Health* 2022; 7:e007116. doi: 10.1136/bmjgh-2021-007116
